# Social context influences the expression of DNA methyltransferase genes in the honeybee

**DOI:** 10.1038/s41598-018-29377-8

**Published:** 2018-07-23

**Authors:** Carlos Antônio Mendes Cardoso-Júnior, Michael Eyer, Benjamin Dainat, Klaus Hartfelder, Vincent Dietemann

**Affiliations:** 1 Departamento de Biologia Celular, Molecular e Bioagentes Patogênicos, Faculdade de Medicina de Ribeirão Preto, Universidade de São Paulo, Avenida Bandeirantes 3900, 14049-900 Ribeirão Preto, SP Brazil; 2 Agroscope, Swiss Bee Research Centre, Schwarzenburgstrasse 161, 3003 Bern, Switzerland

## Abstract

DNA methylation is a reversible epigenetic modification that alters gene expression without altering the nucleotide sequence. Epigenetic modifications have been suggested as crucial mediators between social interactions and gene expression in mammals. However, little is known about the role of DNA methylation in the life cycle of social invertebrates. Recently, honeybees have become an attractive model to study epigenetic processes in social contexts. Although DNA methyltransferase (DNMT) enzymes responsible for DNA methylation are known in this model system, the influence of social stimuli on this process remains largely unexplored. By quantifying the expression of DNMT genes (*dnmt1a*, *dnmt2* and *dnmt3*) under different demographical conditions characterized by the absence or presence of immatures and young adults, we tested whether the social context affected the expression of DNMT genes. The three DNMT genes had their expression altered, indicating that distinct molecular processes were affected by social interactions. These results open avenues for future investigations into regulatory epigenetic mechanisms underlying complex traits in social invertebrates.

## Introduction

Epigenetic modifications are non-genetic mechanisms that regulate gene expression *via cis-* and *trans*-acting factors^1^. DNA methylation occurs through the activity of DNA methyltransferases (DNMTs), which add a methyl radical to the C5 position of cytosines in CpG dinucleotide sequences^2^.

Functional effects of DNA methylation have been investigated intensively over the past years. These research efforts revealed that the methylation process affects multiple functions in animals, such as genomic stability, X chromosome inactivation, embryonic development, cell division, genomic imprinting and responses to environmental changes^1,3,4^. Among others, levels of social interactions affected DNA methylation patterns in mice^5^. Conversely, social behavior of mice after maternal separation-induced stress was also affected by DNA methylation^6^, showing a reciprocal interaction between this molecular process and social traits.

While studied in depth in vertebrates, the interactions between DNA methylation and complex social organization are still poorly studied in invertebrates. Several genes are differentially methylated during caste development in honeybees and in ants^7,8^. For example, the methylation of *egfr* was associated with variation of body size in workers, an important trait affecting social organization in the carpenter ant *Camponotus floridanus*^9^. Functional studies in mammalian and non-mammalian models, also demonstrated that social programming can be modulated after epigenetic impairment^5,10^ and, conversely, that alterations in social stimulation can affect epigenetic modifications^6,11,12^. Hence, studying the molecular regulation of complex social traits in insects might shed light on the evolution of this molecular machinery, which could have been coopted in widely separated taxa with social environments^13,14^.

Honeybee, *Apis mellifera*, colonies contain thousands of individuals interacting as a superorganism^15,16^ and therefore represent an attractive model to study the impact of social context on epigenetic processes. In this species, social context varies according to demographic and seasonal changes^17,18^. In addition, honeybees possess a functional DNA methylation system comprising a complete set of DNMT enzymes^19^. Because DNA methylation is restricted to gene bodies^20^, the honeybee’s methylome size is reduced compared to that of mammals. This likely facilitates the acquisition of direct evidence of its regulatory role on the transcriptome, further supporting the use of honeybees as an excellent model in epigenetics studies. Previous studies linked epigenetic modifications to social aspects of honeybee life history, such as caste differentiation and adult division of labor^8,12,21^, suggesting that DNA methylation functions as mediator between environmental changes and gene expression. However, whether seasonal demographic changes are also reflected in changes in the epigenetic machinery is not yet known.

Recently, alterations in demography that reflect naturally occurring stages of the honeybee colony yearly lifecycle were shown to significantly affect the expression of genes related with aging, endocrine system functions, vitellogenesis and nutritional physiology^22^. Manipulating the presence of young adults and immatures in colonies as in Eyer *et al*.^22^ we here tested the hypothesis that enzymes related with epigenetic processes are involved in the regulatory network underlying the response of honeybee workers to changing social conditions. We monitored the expression of three genes coding for enzymes involved in distinct DNA and RNA methylation mechanisms (*dnmt1a*, *dnmt2* and *dnmt3*), which are known to respond to environmental changes in vertebrates^1^. We expected their expression patterns to vary under the experimentally generated different social contexts. Experimental colonies were kept on the same site and were monitored simultaneously in order to minimize the effect of seasonal and nutritional differences on gene expression. Gene expression was measured over time to take into account age-related physiological and behavioral changes of individual workers^23^. This also allowed gaining insights on the DNMTs expression dynamics. Our results show that the expression of DNMTs encoding genes changes according to alterations in colony demography, and that this differential expression occurs primarily during the first two weeks of a worker’s adult life.

## Material and Methods

### Honeybee source and social manipulations

**Figure 1:**
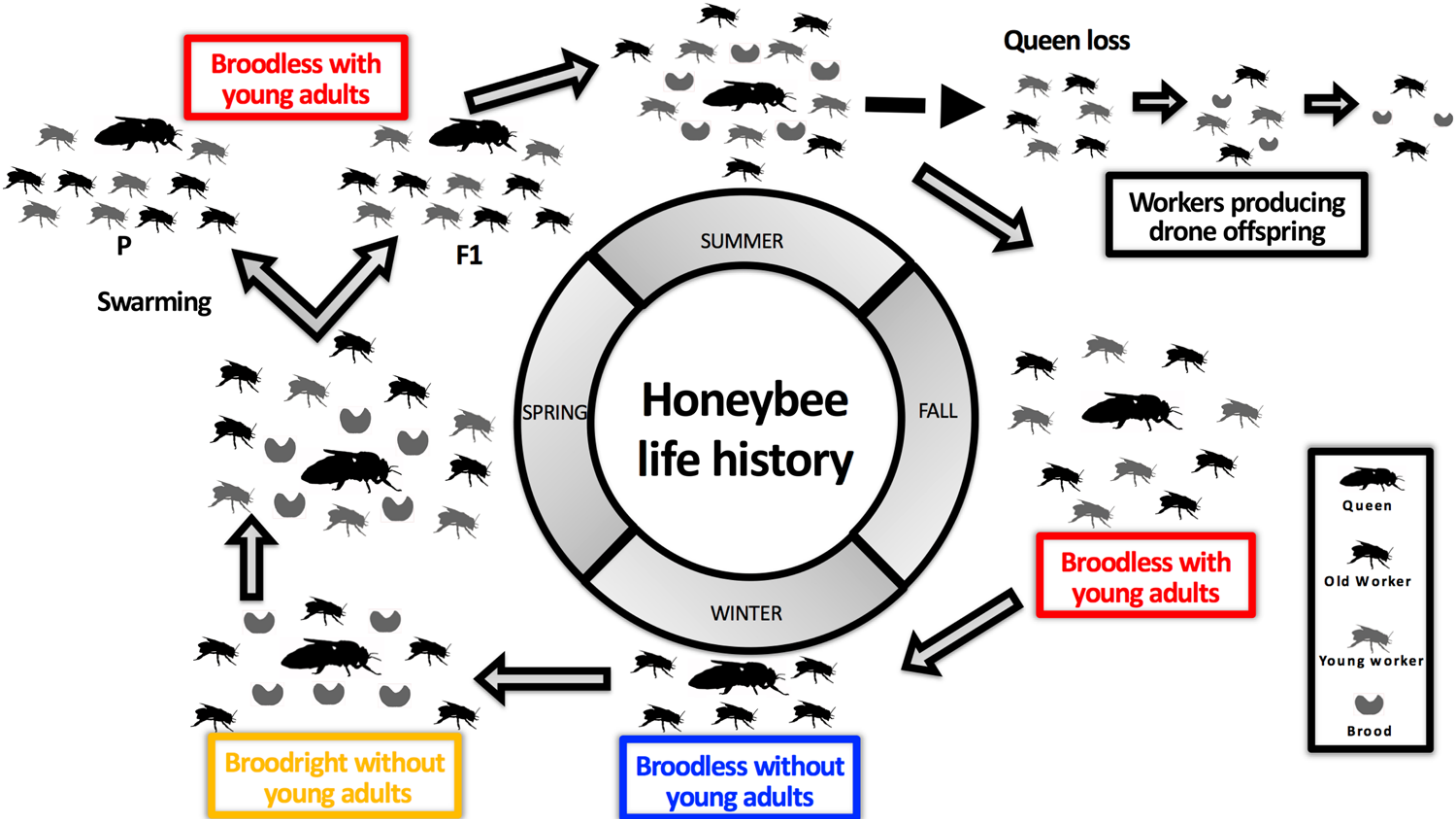
Honey bee life history. In fall, colonies of honeybees contain young adults, old workers and the queen. In winter, brood production is interrupted and colonies only contain the queen and aging workers. When spring comes, queens have started laying eggs, and brood and old workers are both present in the colony, soon joined by young adults emerging from the first brood. Once the colonies have grown sufficiently, reproduction by swarming can occur, and the new colonies (parental and filial – represented as P or F1, respectively) do not contain brood. Brood production starts again after a few weeks and lasts until fall. At any point, colonies can lose their queen, allowing some workers to activate their ovaries and lay unfertilized eggs that develop into drones. If no replacement queen is produced, the colony eventually dies. Large black individuals = queens; small black individuals = old workers; small grey individuals = young adults; small grey comma-shaped individuals = brood. Differently colored boxes represent the social contexts tested in this study.

The honeybees and social manipulations used in this study correspond to those of a previous study^22^, in which we used free flying honeybee (predominantly *A*. *mellifera carnica*) colonies headed by unrelated queens (N = 9, ~16,000 workers each) that were kept in Dadant hives. The experiment was performed from June to August 2013 in Liebefeld, Switzerland. The experimental design consisted of three groups (N = 3 colonies each) displaying different social contexts that naturally occur during the annual life cycle of a honeybee colony (Fig. 1). These social contexts were created by manipulating the presence of immatures (brood) and young adult workers as follow: 1) To generate broodless colonies without young adults, the queens were caged to prevent brood production and young adult emergence during the entire experimental period. 2) The broodless group with young adult workers was generated as for group 1 and by constantly adding newly emerged individuals (N = 450 ± 213) originating from unrelated donor colonies. Additions were performed once daily on 30 occasions during the 35 days-experiment. 3) The broodright group without young adults was created by placing the queens on an empty comb kept in a frame cage, thereby allowing the queens to oviposit on this frame for four days. Subsequently, the combs were removed from the cages and were placed into the same colonies for another nine days, after which they were removed. The repetition of this cycle ensured that open and capped brood was constantly present in the colonies during the experiment but that young adult workers did not emerge.

Prior to experimental manipulation, combs with pupae in late stages of development were incubated at 35 °C and 65% humidity. One day later, 200 newly emerged workers per colony (nine colonies; N = 1,800 individuals) were collected and marked with colored paint dots before being reintroduced into their mother colonies. Five of these marked workers were recaptured from each colony on days 0, 5, 14, 21, 28 and 35 in order to perform gene expression analysis.

### RNA extraction, cDNA synthesis and RT-qPCR assays

**Figure 2:**
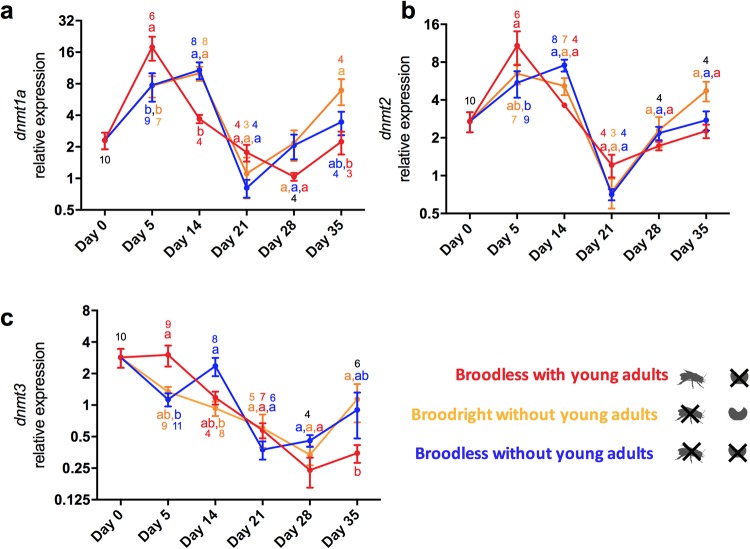
Relative expression of DNA methyltransferase encoding genes in adult honeybee workers of different ages and subjected to different social contexts. Shown are the expression of (**a**) *dnmt1a*, (**b**) *dnmt2* and (**c**) *dnmt3*. Numbers represent the biological sample size and different letters represent statistically significant differences (*p* < 0.05).

The cDNA libraries used in this work were collected during our previous study^22^. Total RNA was extracted from whole bodies of individual workers using a NucleoSpin 96 RNA kit (Macherey-Nagel). For each sample, cDNA was prepared using random hexamer primers, M-MLV Reverse Transcriptase enzyme (Invitrogen^®^) and 10 µL of RNA following the manufacturers’ instructions. Each sample was analyzed twice with a quantitative PCR assay (Kapa SYBR Fast) evaluating the transcript levels of three DNTM genes; *dnmt1a*, *dnmt2* and *dnmt3* using specific primers (see Table 1). The following cycling conditions were used in the RT-qPCR assays: 95 °C for 2 min, 40 cycles of 95 °C for 3 s and 60 °C for 20 s. Simultaneously, the *rp49 (rpl32)* reference gene^24^ was analyzed in the same way. Product specificity was validated for all samples by running a melting curve analysis after the last amplification step. The detection threshold was adjusted manually for each primer set. Relative expression values were calculated using the 2^−∆∆Ct^ equation^25^. The numbers of biological replicates are indicated in Fig. 2.

**Table 1:** Primer sequences used in the RT-qPCR assays.

Name	Sequence 5′ 3′	Reference	Beebase access code
DNMT1a F	CGAGTAGTAAGCGTGCGTGAA	Cardoso-Junior, *et al*. 2017	GB47348
DNMT1a R	CAAGTGGTGGAGGAACTGC		
DNMT2 F	TGAGTCCTCCATGTCAACCTT	Biergans, *et al*. 2015	GB54141
DNMT2 R	GCCAAATTGACAAGGGCTTA		
DNMT3 F	CAGCGATGACCTGCGATCGGCGATA	Lockett, *et al*. 2010	GB55485
DNMT3 R	TACAGGG TTTATATCGTTCCGAAC		
RP49 F	CGTCATATGTTGCCAACTGGT	Lourenço, *et al*. 2008	GB47227
RP49 R	TTGAGCACGTTCAACAATGG		

Shown are the name of the genes, the respective F (Forward) and R (Reverse) primer sequences, the primer references, and the beebase database access codes.

### Statistical analysis

Log^10^ transformed data was shown to be normally distributed with the Kolmogorov-Smirnov test. We thus used a two-way ANOVA and a Tukey-Kramer *post-hoc* test with correction for multiple comparisons to compare the gene expression of workers under the three social conditions at each time-point (Table 2). A *p*-value < 0.05 was considered significant. All analyses were performed with the software GraphPad Prism version 5.0.

**Table 2:** Statistical analysis details for the expression of the DNMTs encoding genes.

Test	Day	Source of variation or Multiple comparisons	Genes		
			*dnmt1a*	*dnmt2*	*dnmt3*
Two-Way ANOVA	—	Social context	F_(2,88)_ = 1.02; *p = 0*.*3647*	F_(2,88)_ = 0.07346; *p* = *0*.*9292*	F_(2,109)_ = 0.2761; *p* = *0*.*7593*
	—	Time	F_(5,88)_ = 27.9; *p* < *0*.*0001*	F_(5, 88)_ = 29.65; *p* < *0*.*0001*	F_(5,109)_ = 30.75; *p* < *0*.*0001*
	—	Interaction between ‘social context’ and ‘time'	F_(10,88)_ = 3.216; *p* = *0*.*0014*	F_(10,88)_ = 1.755; *p* = *0*.*081*	F_(10,109)_ = 2.574; *p* = *0*.*0078*
Tukey-Kramer post-hoc test	5	Broodless with young adults vs broodless without young adults	*p* = *0*.*0084*	*p* = *0*.*0481*	*p* = *0*.*0093*
		Broodless with young adults vs broodright without young adults	*p* = *0*.*0469*	*p* = *0*.*3979*	*p* = *0*.*0853*
		Broodless without young adults vs broodright without young adults	*p* = *0*.*8576*	*p* = *0*.*5334*	*p* = *0*.*7353*
	14	Broodless with young adults vs broodless without young adults	*p* = *0*.*0238*	*p* = *0*.*077*	*p = 0*.*3829*
		Broodless with young adults vs broodright without young adults	*p* = *0*.*0381*	*p* = *0*.*6805*	*p* = *0*.*7266*
		Broodless without young adults vs broodright without young adults	*p* = *0*.*9729*	*p* = *0*.*2569*	*p = 0*.*0315*
	21	Broodless with young adults vs broodless without young adults	*p* = *0*.*1558*	*p* = *0*.*3666*	*p* = *0*.*3852*
		Broodless with young adults vs broodright without young adults	*p* = *0*.*4044*	*p* = *0*.*3946*	*p* = *0*.*9949*
		Broodless without young adults vs broodright without young adults	*p* = *0*.*9017*	*p* = *0*.*9987*	*p = 0*.*502*
	28	Broodless with young adults vs broodless without young adults	*p* = *0*.*3495*	*p* = *0*.*817*	*p* = *0*.*1895*
		Broodless with young adults vs broodright without young adults	*p* = *0*.*4291*	*p* = *0*.*9286*	*p* = *0*.*638*
		Broodless without young adults vs broodright without young adults	*p* = *0*.*9885*	*p* = *0*.*9689*	*p* = *0*.*6728*
	35	Broodless with young adults vs broodless without young adults	*p* = *0*.*3437*	*p* = *0*.*8725*	*p* = *0*.*1729*
		Broodless with young adults vs broodright without young adults	*p* = *0*.*019*	*p* = *0*.*1253*	*p* = *0*.*0215*
		Broodless without young adults vs broodright without young adults	*p* = *0*.*3113*	*p* = *0*.*3085*	*p* = *0*.*6417*

Shown are the results for the Two-Way ANOVA and Tukey-Kramer *post-hoc* tests, including the *F* value of the ANOVA test and adjusted *p*-values for source of variation and multiple comparison analysis. Significant differences (*p* < 0.05) are represented in bold.

## Results

Expression of the three DNMT genes did not differ significantly between social contexts over the whole experimental period, but varried significantly over time (Table 2). In addition, the interaction between social context and time was statistically significant for *dnmt1a* and *dnmt3* expression but not for *dnmt2* (Table 2). The RT-qPCR analyses performed over 35 days, which cover most of the lifespan of an adult worker in summer, revealed that the expression levels of *dnmt1a* were significantly higher at day 5 in workers from colonies with young adults, when compared with the two groups lacking young adults (Fig. 2a). At day 14, the expression of *dnmt1a* was significantly lower in the group with young adults compared with the two groups lacking young adults (Fig. 2a). As for day 5, no statistical difference was found between the broodright and broodless groups lacking young adults (Table 2). For 21 and 28 days-old individuals, gene expression was not significantly different between any of the groups (Table 2). At day 35, a statistically significant difference was found between the broodless group with young adults and the broodright group lacking young adults, but not between the broodless groups either with, or without young adults (Table 2). Moreover, no statistical difference was found between the broodright and broodless groups lacking young adults.

The expression pattern of the RNA methyltransferase *dnmt2* gene over time was similar to that of *dnmt1a* (Fig. 2b) but showed fewer time points with significant differences between the groups. The only difference was observed at day 5, when gene expression was statistically higher in broodless colonies containing young adults compared to broodless colonies lacking young adults (Table 2).

The expression of *dnmt3* was significantly higher in 5-day-old individuals from broodless colonies with young adults than in individuals from broodless colonies lacking young adults. In contrast, there was no significant difference in expression compared to individuals from broodright colonies without young adults (Fig. 2c). Once more, no statistical difference was found between the broodright and broodless groups without young adults (Table 2). At day 14, *dnmt3* expression was statistically different between individuals from broodright and from broodless colonies lacking young adults. No statistical differences were observed for the other combinations of groups. For individuals aged 21 and 28 days, gene expression was not significantly different between any of the groups. At day 35, individuals from broodless colonies with young adults showed a significantly lower expression of *dnmt3* than individuals from broodright colonies lacking young adults. No difference was observed for the other combinations of groups (Table 2).

## Discussion

Gene expression has been shown to be modulated by reversible epigenetic marks in different animal models^26^. Here, we show for the first time an effect of social context on the epigenetic machinery in the honeybee. Hence, our results suggest a role of epigenetic mechanisms as mediators of adaptive responses to social environment.

In this study, we focused on the effect of social context on the expression of three genes belonging to distinct classes of nucleotide modifiers^1^. Our results imply that DNA methylation can be activated by brood and young adults, unlike RNA methylation that seems to be only affected by the presence of young adults. These data, together with the fact that Hymenopterans show dynamic methylome process^12,27,28^ possibly involving DNMTs mRNA levels^29^, suggest that activation of different epigenetic pathways appears necessary to fine-tune the levels of effector proteins required by honeybees to adapt to their social context.

Expression of all the DNMTs encoding genes tested here was variable during the adult life of workers. This finding is in accordance with previous studies in honeybees and other social insects that reported changes in DNMT expression and/or DNA methylation levels involved in the regulation of multiple processes, such as development, aging, behavior and reproduction^12,21,27,30–32^. Although, social context alone was not a significant source of variance in the expression of *dnmts*, their interaction with time affected *dnmt1a* and *dnmt3* expression. This indicates that social context affects the expression of these genes differently at specific time points. Large differences in expression were observed during the first days of adult life, suggesting that honeybee workers can quickly respond to social stimuli that are likely to regulate their behavioral maturation. We found that the presence of young adults affected the expression of all three DNMTs encoding genes early in the adult life cycle, whereas the presence of brood seemed to only affect the expression of *dnmt3* at day 14. A possible explanation for this pattern is that social manipulation accelerated the behavioral maturation dynamics of nestmates, making them age faster. This hypothesis is supported by our previous study^22^, which showed that the presence of young adults and brood altered the expression of aging-related genes and survival rates in their nestmates. The *dnmt1a* and *dnmt3* transcript levels were also different between 35 days-old workers from broodless colonies that contained young adults and from broodright colonies without young adult colonies. The ability to respond to external changes is, thus, not restricted to the beginning of adult life, but can also be expressed at later stages, when the workers have typically started foraging^33^.

DNA methylation was previously associated with different degrees of behavioral maturation in honeybees, whereby distinct patterns of DNA methylation were shown to be associated with workers age polyethism, *i*.*e*., the transition from within-colony to foraging task performance^12^. These patterns were found to be reversible and contingent on the respective social task. Our results support the hypothesis that social environments, here represented by demographic changes related to the presence of brood and young adults, alter nucleotide methylation that may mediate behavioral maturation networks. For example, the expression of *dnmt1a*, an enzyme necessary for maintenance of global DNA methylation levels, peaked at day 5 in the group with young adults, but was delayed until day 14 in workers from colonies lacking young adults, independently of whether these colonies had brood or not. This peak may promote distinct genomic methylation patterns that drive alterations in gene activity and ultimately, worker behavior and aging. Furthermore, *dnmt2* expression, which was upregulated at day 5 in the group with young adults, has been associated with tRNA protection against ribonuclease cleavage and microRNA biogenesis following environmental stress in *Drosophila melanogaster*^34,35^. Thus, DNMT2 seems to act downstream of gene expression by regulating microRNA decay. On the same day (5), *dnmt3* expression also exhibited a peak in this group (with young adults). Interestingly, variation in *dnmt3* expression, a *de novo* DNA methyltransferase, was shown to affect aging and alternative splicing in honeybee workers^29,31^, thus suggesting that fluctuations in *dnmt3* transcript levels may represent a molecular signature of behavioral maturation through mRNA regulation. Therefore, it is possible that the expression of all three DNMTs encoding genes studied here is sensitive to external stimuli, especially early in a worker’s adult life. Consequently, the resulting epigenetic modifications might influence gene regulatory networks associated with the workers behavioral ontogeny in colonies containing young adult workers.

We found that the presence of brood affected *dnmt3* expression. This effect is possibly mediated through a brood pheromone^36^. A recent study showed that *dnmt3* gene expression is also affected by a pheromone produced in the mandibular gland of *A*. *mellifera* queens^37^. This pheromone spreads within the colony and signals the presence of the queen and influences worker behavior^38^. Through their pheromonal signals, queens and brood, thus, appear to affect the same epigenetic component in the regulatory circuitry underlying behavioral plasticity of workers.

Finally, our results provide empirical evidence for an epigenetic response to seasonal demographic variation in honeybee colonies that is in line with previous studies^10,12,21^. The results of these studies, together with ours, highlight the important role of DNA methylation in insect societies, acting in processes that are analogs to those described in mammals^5,6,11^. This indicates that social insects may make use of an evolutionarily conserved epigenetic machinery to modulate behavioral responses according to social context.

Although we herein focused on the social context resulting from alterations in colony demography, our results shed light on social interactions at the molecular level in general, thus expanding the available knowledge on epigenetic mechanisms of complex traits seen in social insects. Future investigations will be necessary to elucidate the possible roles of DNMTs and DNA methylation in other socially regulated processes, such as hygienic behavior, worker policing, aggressiveness, and regulation of reproduction.
