# Publisher Correction: Social context influences the expression of DNA methyltransferase genes in the honeybee

**DOI:** 10.1038/s41598-018-32087-w

**Published:** 2018-09-26

**Authors:** Carlos Antônio Mendes Cardoso-Júnior, Michael Eyer, Benjamin Dainat, Klaus Hartfelder, Vincent Dietemann

**Affiliations:** 1 Departamento de Biologia Celular, Molecular e Bioagentes Patogênicos, Faculdade de Medicina de Ribeirão Preto, Universidade de São Paulo, Avenida Bandeirantes 3900, 14049-900 Ribeirão Preto, SP Brazil; 2 Agroscope, Swiss Bee Research Centre, Schwarzenburgstrasse 161, 3003 Bern, Switzerland

Correction to: *Scientific Reports* 10.1038/s41598-018-29377-8, published online 23 July 2018

This Article contains formatting errors in Table 2. The correct Table 2 appears below, with p values < 0.05 highlighted in bold.

**Table 2:** Statistical analysis details for the expression of the DNMTs encoding genes. Shown are the results for the Two-Way ANOVA and Tukey-Kramer *post-hoc* tests, including the *F* value of the ANOVA test and adjusted *p*-values for source of variation and multiple comparison analysis. Significant differences (*p* < 0.05) are represented in bold.

Test	Day		Genes		
		Source of variation or Multiple comparisons	*dnmt1a*	*dnmt2*	*dnmt3*
Two-Way ANOVA	—	Social context	F_(2,88)_ = 1.02; *p* = *0.3647*	F_(2,88)_ = 0.07346; *p* = *0.9292*	F_(2,109)_ = 0.2761; *p* = *0.7593*
	—	Time	**F**_**(5,88)**_ = **27.9**; ***p*** < ***0.0001***	**F**_**(5, 88)**_ = **29.65**; ***p*** < ***0.0001***	**F**_**(5,109)**_ = **30.75**; ***p*** < ***0.0001***
	—	Interaction between ‘social context’ and ‘time’	**F**_**(10,88)**_ = **3.216**; ***p*** = ***0***.***0014***	F_(10,88)_ = 1.755; *p* = *0.081*	**F**_**(10,109)**_ = **2.574**; ***p*** = ***0***.***0078***
Tukey-Kramer post-hoc test	Day 5	Broodless with young adults vs broodless without young adults	***p*** = ***0***.***0084***	***p*** = ***0***.***0481***	***p*** = ***0.0093***
		Broodless with young adults vs broodright without young adults	***p*** = ***0***.***0469***	*p* = *0.3979*	*p* = *0.0853*
		Broodless without young adults vs broodright without young adults	*p* = *0.8576*	*p* = *0.5334*	*p* = *0.7353*
	Day 14	Broodless with young adults vs broodless without young adults	***p*** = ***0***.***0238***	*p* = *0.077*	*p* = *0.3829*
		Broodless with young adults vs broodright without young adults	***p*** = ***0***.***0381***	*p* = *0.6805*	*p* = *0.7266*
		Broodless without young adults vs broodright without young adults	*p* = *0.9729*	*p* = *0.2569*	***p*** = ***0.0315***
	Day 21	Broodless with young adults vs broodless without young adults	*p* = *0.1558*	*p* = *0.3666*	*p* = *0.3852*
		Broodless with young adults vs broodright without young adults	*p* = *0.4044*	*p* = *0.3946*	*p* = *0.9949*
		Broodless without young adults vs broodright without young adults	*p* = *0.9017*	*p* = *0.9987*	*p* = *0.502*
	Day 28	Broodless with young adults vs broodless without young adults	*p* = *0.3495*	*p* = *0.817*	*p* = *0.1895*
		Broodless with young adults vs broodright without young adults	*p* = *0.4291*	*p* = *0.9286*	*p* = *0.638*
		Broodless without young adults vs broodright without young adults	*p* = *0.9885*	*p* = *0.9689*	*p* = *0.6728*
	Day 35	Broodless with young adults vs broodless without young adults	*p* = *0.3437*	*p* = *0.8725*	*p* = *0.1729*
		Broodless with young adults vs broodright without young adults	***p*** = ***0***.***019***	*p* = *0.1253*	***p*** = ***0***.***0215***
		Broodless without young adults vs broodright without young adults	*p* = *0.3113*	*p* = *0.3085*	*p* = *0.6417*

